# An update on novel approaches for diagnosis and treatment of SARS-CoV-2 infection

**DOI:** 10.1186/s13578-021-00674-6

**Published:** 2021-08-22

**Authors:** Azadeh Safarchi, Shadma Fatima, Zahra Ayati, Fatemeh Vafaee

**Affiliations:** 1 School of Biotechnology and Biomolecular Science, University of New South Wales, NSW Sydney, Australia; 2 Ingham Institute of Applied Medical Research, Liverpool, Australia; 3 Department of Traditional Pharmacy, School of Pharmacy, Mashhad University of Medical Sciences, Mashhad, Iran; 4 NICM Health Research Institute, Western Sydney University, Penrith, Australia; 5 UNSW Data Science Hub University of New South Wales, NSW Sydney, Australia

**Keywords:** COVID-19, SARS-COV-2, Diagnostics, Vaccines, Treatment

## Abstract

The ongoing pandemic of coronavirus disease 2019 (COVID-19) has made a serious public health and economic crisis worldwide which united global efforts to develop rapid, precise, and cost-efficient diagnostics, vaccines, and therapeutics. Numerous multi-disciplinary studies and techniques have been designed to investigate and develop various approaches to help frontline health workers, policymakers, and populations to overcome the disease. While these techniques have been reviewed within individual disciplines, it is now timely to provide a cross-disciplinary overview of novel diagnostic and therapeutic approaches summarizing complementary efforts across multiple fields of research and technology. Accordingly, we reviewed and summarized various advanced novel approaches used for diagnosis and treatment of COVID-19 to help researchers across diverse disciplines on their prioritization of resources for research and development and to give them better a picture of the latest techniques. These include artificial intelligence, nano-based, CRISPR-based, and mass spectrometry technologies as well as neutralizing factors and traditional medicines. We also reviewed new approaches for vaccine development and developed a dashboard to provide frequent updates on the current and future approved vaccines.

**Supplementary Information:**

The online version contains supplementary material available at 10.1186/s13578-021-00674-6.

## Background

**Fig. 1:**
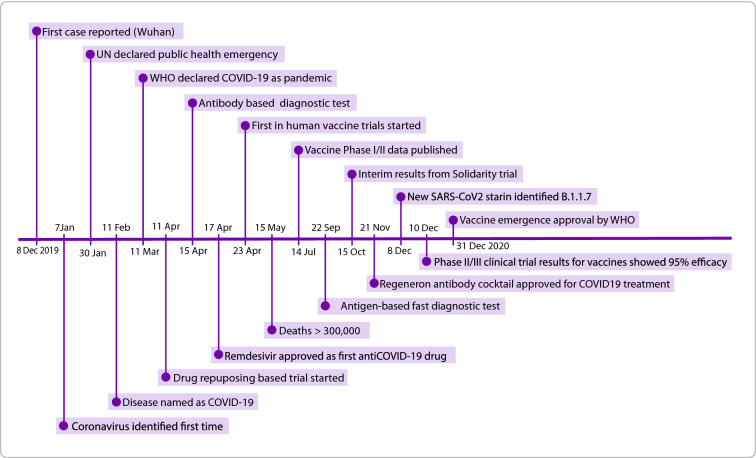
Timeline showing major events regarding COVID-19 outbreak from 2019 to 2020

As of late June 2021, with over 180 million confirmed cases and more than 3.9 million deaths especially in the elderly generation (https://covid19.who.int/), the whole world is still struggling with the infectious viral disease called COVID-19 caused by SARS-CoV-2. A brief timeline of events related to COVID-19 is depicted in Fig. 1. Like other coronaviruses, SARS-CoV-2 is an enveloped virus with a large positive single strand RNA genome (around 30 kb) [1]. The genome has segments and multiple open reading frames (ORFs) encoding non-structural and structural proteins namely spike protein (S), envelope protein (E), membrane protein (M), and nucleocapsid protein (N) [2, 3]. The virus first infects the respiratory tract and then other organs via the attachment of homotrimer spike through receptor-binding domain (RBD) to its host receptors including angiotensin converting enzyme 2 (ACE2), transmembrane protease, serine 2 (TMPRSS2), cathepsin L/B (CTSL/B, and dipeptidyl peptidase 4 (CD26) [4, 5]. It has been shown that the human receptors, particularly ACE2 and TMPRSS2, are the cell surface proteins of epithelial cells of lungs, small intestines, vascular endothelial cells, kidneys, heart, and cortical neurons with different expression level based on the age, gender, and smoking conditions as well as very low expression in infants and toddlers [6, 7]. A recent preprint study calculated 7.4 ± 3.4 (mean ± SD) mutations per genome for the virus and by analyzing 261,323 full-length SARS-CoV-2 global genomes showed that the majority of current predominant variants of SARS-CoV-2 in the world were derived from an M type mutant with concurrent mutation of T8782C (ORF1ab) and C28144T (ORF8) that initially collected from the Market in Wuhan, China [8]. Furthermore, a SARS-CoV-2 variant with a nonsynonymous mutation A23403G (D614G) in the spike protein emerged during the first months of pandemic, February 2020, with a better fitness and higher infectivity [9, 10]. A fast spreading variant called Variant of Concern 202012/01 (VOC-202012/01; also known as B.1.1.7 or variant Alpha) has been emerged with 17 mutations of which eight mutations located in the spike including two notable mutations (N501Y, P681H) and two deletions (H69, V70) [11]. There are also ongoing new variants spreading in different countries listed by WHO (https://www.who.int/en/activities/tracking-SARS-CoV-2-variants/) of which some have high transmissibility including the variants reported firstly in South Africa (B.1.351or variant Beta) and Brazil (P.1 or variant Gamma) that share the N501Y mutation, variant (B.1.617.2 known as variant Delta) in India that has two mutations as E484Q and L452R. The ongoing genomic changes in the SARS-CoV2 genome has increased concerns in terms of false negative results in diagnosis, better fitness and higher transmissibility and infectivity of the virus and may decrease the vaccine efficacy in the population [12–15].

Most infected individuals develop an asymptomatic to mild form of the disease, with symptoms varying from headache and fatigue to fever and dry cough [16]. Asymptomatic infection has been reported at any age but are most frequent in younger population especially in children [17]. Less than 10% of patients might develop diffuse alveolar damage (DAD) that might lead to acute respiratory distress syndrome (ARDS) [18]. Other multiorgan complications especially among hospitalized patients include acute liver injury, cardiac injury, acute heart failure, acute kidney injury, and gastrointestinal symptoms like nausea, vomiting or diarrhea [19].

Early diagnosis is a key role to stop the spread. Currently reverse-transcriptase polymerase chain reaction (RT-PCR) is a gold standard to detect the SARS-CoV-2 sequence in samples mainly taken from nasopharynx of suspected individuals [20]. The target genes may differ, but mostly include N1, N2, E, S, ORF1ab and the RNA polymerase gene, RdRP, of which at least two genes need to be detected using single or multiplex RT-PCR in combination with Human RNase P as an internal control [20, 21]. Combination diagnosis including the RT-PCR and the additional investigation using computed tomography (CT) scan can enhance the sensitivity of the disease detection [22].

Several point of care testing (POC) methods have been developed that can detect the presence of viral nucleic acid, antigen or antibodies including Immunoglobulins G (IgG), M (IgM) and A (IgA) within a few hours [23]. These include kits mostly based on loop-mediated isothermal amplification (LAMP), immunochromatographic assay, chemiluminescent immunoassay (CLIA), microfluidic immunofluorescence assay, enzyme-linked immunosorbent assay (ELISA), and lateral flow immunoassay (LFIA). These diagnostic tests are detailed in previous reviews [23, 24] and summarized in Table 1

**Table 1:** Most current available diagnostic tests for SARS-CoV 2

Approach	Detection	Mechanisms	Advantages	Disadvantages
RT-PCR	Different genes (S, E, N, RdRp, ORF1ab)	Reverse transcriptase PCR amplification (thermal cycles)	High sensitivity and specificity with low copy number of virus	False +/−, qualified technician, expensive, only in laboratory, only for active infection, uncomfortable swab sampling
LAMP, RT-LAMP, RPA	Different genes (S, E, N, RdRp, ORF1ab)	PCR amplification (without thermal cycle)	Rapid screening (POC), time saving, user friendly, simple equipment	Low sensitivity, False +, primary validation, only for active infection, uncomfortable swab sampling
CRISPR/cas	Different genes (S, E, N, RdRp, ORF1ab)	Detection and cleavages of viral RNA by CRISPR-cas9, 12, 13 systems	Rapid screening (POC), time saving, user friendly, high specificity and sensitivity, read results by naked eye or simple instrument	Low sensitivity due to viral adaptation, high cost
ELISA	Viral Antigens (proteins)/antibodies	Detection of Ag/Ab in the sample based on the attachment of anti-Ag/Ab and florescent visualization	Quantitative detection, stable reagent, high sensitivity for Ab detection, can be visualized by using Au nanoparticles, low cost	Less accuracy and low sensitivity especially in Ag detection at later phase of the disease, time consuming, high cost, difficult for early diagnosis in case of Ab detection
LFIA	Viral antigens (proteins)/antibodies	Detection of Ag or Ab in the plasma based on the attachment of antiAG/Ab on the nitrocellulose membrane and nanoparticle visualization	Rapid screening (POC) and time saving, simple and user friendly, read the results by digital instrument or naked eye	Less accuracy and low sensitivity, not suitable for early diagnosis, false nagative, verification needed
CLIA	Viral antigens (proteins)/antibodies	Detection of Ag or Ab in the plasma based on the attachment of anti-Ag/Ab on the magnetic, protein-coated microparticles and visualization by chemiluminescent	Rapid screening (POC) and time saving, automated instruments	High cost, not suitable for early diagnosis, need supporting chemiluminescence instruments

Test are based on the detection of viral genes or proteins (Ag) or the presence of Antibodies in the patient’s sample that are mostly nasopharynx swabs or blood (plasma)

*LAMP* loop-mediated isothermal amplification, *POC* point of care, *ELISA* enzyme linked immunosorbent assay, *LFIA* lateral flow immunoassay, *CLIA* chemiluminescent immunoassay; more details available at Nguyen et al. [136] and Kubina et al. [137]

Several clinical trials are investigating the efficacy of the novel COVID-19 therapeutics or the existing repurposed medications including antivirals such as remdesivir, favipiravir and umifenovir that have been previously used to control ebola and influenza, of which remdesivir was recently approved by FDA for emergency use to treat COVID-19 for hospitalized patients aged 12 years and older [16, 25]. Furthermore, other therapeutics including anti-inflammatory medicines (e.g., dexamethasone, methylprednisolone), monoclonal and polyclonal antibodies (e.g., Regenin), convalescent plasma, immunomodulators (e.g., Interferon-β-1a and Tocilizumab) have been proposed and is currently prescribed (see Additional file 1: Table S1) and ongoing clinical trials are investigating their effects on managing the disease [16, 25, 26].

**Fig. 2:**
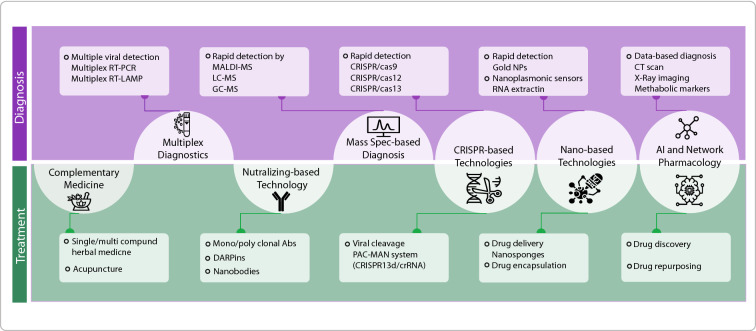
Schematic summary of novel diagnostic and therapeutic approaches for COVID-19. The focus has been on interdisciplinary approaches of which some techniques such as CRISPR-based, nano-based technologies and AI are used in both diagnostics and therapeutic approaches. Simultaneous detection of SARS-CoV-2 and influenza virus A and B by multiplex RT-PCR and RT-LAMP as well as mass spectrometry-based techniques including matrix-assisted laser desorption/ionization (MALDI-MS), liquid chromatography spectrometry (LC–MS) and gas chromatography spectrometry (GC–MS) were also reviewed in diagnostic approaches. New treatment platforms for neutralizing agents such as mono and poly clonal antibodies, nanobodies and designed ankyrin repeat proteins (DARPines) as well as complementary medicine have been discussed

In addition to the currently approved diagnostic techniques, nominated drugs, and proposed vaccines that are under latest phases of clinical trials, there are various novel and innovative approaches focusing on the rapid and accurate diagnosis and treatment of COVID-19. These techniques can help health policymakers, researchers, and communities to mitigate the effect of the COVID-19 pandemic in the world and develop capacities for the management of possible emerging infections in the future. Here, we first review novel and multidisciplinary approaches for diagnosis of the disease and then, focus on the potential interdisciplinary approaches towards novel medicine and vaccines COVID-19 (Fig. 2).

## Diagnosis

Current diagnostic tests have their own limitations including time, specificity, technician training, and cost. Here, we discuss some fast and accurate biomolecular approaches based on the latest technologies that have been suggested, developed, and even approved to be used by clinical laboratories.

### Multiplex real-time PCR technology

Due to the similar presentation of COVID-19 and influenza, designing diagnostic approaches that can simultaneously detect multiple viruses in the patient is beneficial, cost and time saving. Multiplex reverse transcription-polymerase chain reaction (RT-PCR) assay for detecting SARS-COV-2 and influenza concurrently can reduce reagents, time, and potential human error per sample. Norz et al. developed a multiplex RT-PCR assay that simultaneously detects SARS-CoV-2, influenza A and influenza B viruses with respective sensitivity of 98.1%, 97.7%, and 100% for each virus. Four set of primer/probes for E and RdRP genes of SARS-CoV-2, M gene of influenza A, and NS2 gene of influenza B were adapted and modified with 2′*O*-methylated RNA-bases at their penultimate (3′-end) positions to reduce formation of primer dimers [27]. In another study by Mancini et al., primers for influenza virus A and B, N2 and E genes of SARS-CoV-2, and the human RP gene as internal control, were used to simultaneously detect the viruses in the multiplex RT-PCR assay in 1000 clinical samples of which two coinfections of SARS-CoV-2 and influenzas were reported [28]. Moreover, the USA Centers for Disease Control and Prevention (CDC) developed the Influenza SARS-CoV-2 (Flu SC2) multiplex RT-PCR assay that was received Emergency Use Authorization (EUA) approval by FDA. The Flu SC2 diagnostic kits contain primers and probs for N gene of SARS-CoV-2, M1 gene of influenza A, and NS2 gene of influenza B along with PR gene of human as internal control [21]. Furthermore, Zhang and Tanner developed a multiplex reverse transcription loop-mediated isothermal amplification (RT-LAMP) assay with high sensitivity for the simultaneous point-of-care testing of SARS-CoV-2 and Influenza A and B. In RT-LAMP, the amplification occurs at a constant temperature of 60–65 °C without thermal cycling which makes it a rapid and sensitive diagnostic technique [29].

### CRISPR-based technology

One of the biomolecular approaches introduced to the market for a rapid (less than an hour) diagnosis of COVID-19 is based on the CRISPR-cas nucleic acid editing technology. CRISPR (Clustered Regulatory Interspersed Short Palindromic Repeats) systems were first discovered in *E. coli* in 1987 and later in other species [30]. It is based on the generating specific CRISPR RNA (crRNA) which target invasive RNA/DNA sequences and cleave it into multiple smaller sequences by the endonuclease activity of CRISPR-associated (cas) proteins [31]. Currently, several highly sensitive CRISPR-cas based tests were introduced for the rapid and accurate detection of SARS-CoV-2. These tests are based on various CRISPR-cas types including Fncas9 (FELUDA), cas12a (AIOD-CRISOR and DETECTR), and cas13 (SHERLOK and CREST) and detect different parts of the viral genome such as E, N2, S and ORF1ab genes. DETECTR (DNA Endonuclease Targeted CRISPR Trans Reporter) and SHERLOK (Specific High-sensitivity Enzymatic Reporter UnLOCKing) have been approved by FDA under EUA [30, 32–36]. In these methods, viral RNA is first purified and amplified followed by the substrate cleavage of the RNA/DNA complex with CRISPR-cas proteins. The results can be visualized by paper flow strips, agarose gel, or fluorescent markers. A recent study published in January 2021, introduces a novel amplification-free CRISPR-Cas13a assay for a rapid and accurate detection of SARS-CoV-2 RNA in patient samples that can be read with a mobile phone microscope allowing portable readout [37]. The assay achieved (100 copies/ml) sensitivity in under 30 min and identified a set of SARS-CoV-2-positive patient RNA samples within 5 min. Unlike previous methods that relies on the pre-amplification of viral RNA, this assay directly detects the viral genome by combining multiple SARS-CoV-2 CRISPR-DNA (crDNA) that bind to the target RNA, resulting the activation of Cas13a protein. The protein cleaves a quenched-fluorophore RNA reporter, allowing for fluorescence detection as a proxy for Cas13a activation and target RNA; the florescent can be simply measured by a mobile phone camera in a compact device [37].

### Nano-based technology

Numerous studies investigated the possible use of nano-based biosensors including electrochemical, optic, piezoelectric, thermal and magnetic-based nano sensors, for the detection of viral pathogen or antibodies [38]. Different approaches and nano-based kits have been introduced to detect SARS-CoV-2 or related antibodies using gold nanoparticles (AuNPs) due to their unique photonic, electric, and catalytic features which have enabled them to specifically couple with various biomarkers like antibodies or nucleic acids [38]. Moitra et al. [39] have developed an AuNP-based colorimetric assay that capped with thiol-modified antisense oligonucleotides (ASO) specifically designed for N gene and can detect the presence of SARS-CoV-2 from the isolated RNA samples within 10 min. In this method, the ASO capped with AuNP agglomerates selectively in the presence of N gene and shows a change in its surface plasmon resonance. Moreover, field-effect transistor (FET)-based biosensors—i.e., graphene sheets of FET coated with a specific anti-spike protein antibody were developed that can detect the low concentration of SARS-CoV-2 spike protein [40]. A rapid and directly optical measurement of SARS-CoV-2 particle was also suggested by using a spike protein specific nanoplasmonic resonance sensor. The results are attainable within 15 min and can be visualized on generic microplate reader and a handheld smartphone connected device that decrease the cost and time of virus detection [41]. Zhao et al. [42] designed an RNA extraction method based on poly carboxyl groups-coated magnetic nanoparticles (pcMNP) that combines the virus lysis and RNA binding steps into a single step to enhance the time (30 min) and sensitivity of the RNA extraction.

### Mass spectrometry-based technology

Mass spectrometry (MS) is based on ionization of chemical molecules including biological proteins and measuring their mass charges. MS technologies have led to the development of diagnostic approaches especially after the introduction of electrospray ionization (ESI) and matrix-assisted laser desorption/ionization (MALDI) techniques that can obtain ions from large molecules with minimal fragmentation [43]. Identification of microorganisms by MALDI is based on either peptide mass fingerprints (PMF) or matching the masses of biomarkers of unknown organism with the proteome databases [44]. So far, numerous studies have investigated and developed MALDI-based techniques for laboratory diagnosis of HPV, HBV, and HBC due to its rapid, low cost, low detection limit and high accuracy properties [43, 44]. Recently, an MALDI-MS-based approach along with machine learning analysis was developed for detection of SARS-CoV-2 using impurified nasal swab specimens with minimal specimen preparation, few reagents, flexible sample number and rapid data acquisition [45]. Swab samples in the transfer media were directly applied to the MALDI-MS and results were analyzed by six different machine learning approaches and authors claimed to have results with high accuracy (93.9%), low false positive (7%), and false negative (5%) rates. In another study by Schuster et al*.* a rapid SARS-CoV-2 identification was developed based on liquid chromatography-MS using six specific and sensitive SARS-CoV-2 peptide markers [46]. It is claimed that the assay can identify low concentration (10^4^ PFU/ml) of SARS-CoV-2 in naïve nasopharyngeal swabs. Furthermore, it is suggested to use MS-based approaches to detect host immune responses and predict the severity of COVID-19 within the infected individuals to enhance the management of the disease symptoms [47].

Various MS-based techniques are used to develop breath analyzer devices that can distinguish suspected COVID-19 patients based on the panel of volatile compounds in their breath as per a survey by the International Federation of Clinical Chemistry (IFCC), August 2020 [48]. A group of researchers in Germany and UK developed a method to detect COVID-19 patients by the analysis of breath using gas chromatography-ion mobility spectrometry (GC-IMS) with the accuracy of 80% [49]. It is suggested that a biomarker panel of volatile organic compounds (e.g., ethanal, octanal, acetone, butanone, methanol, heptanal) provides the basis of a COVID-19 rule- in/rule-out breath-test. In another study, proton-transfer-reaction quadrupole time-of-flight mass spectrometer was for analysis of exhaled breath of COVID-19 ARDS patients and found four prominent volatile compounds of methylpent-2-enal, 2,4-octadine 1-chloroheptane and nonanal [50]. These studies can help first healthcare contacts to rapidly distinguish suspected patients and their disease stages and proceed further investigation faster.

### AI-assisted COVID-19 diagnosis

The availability of large-scale, multi-sourced and real-time patient clinical information, medical imaging data and high-throughput genomic or protein information, machine learning (ML) and artificial intelligence (AI) algorithms have become powerful techniques enabling reliable pre-clinical screening and accurate clinical diagnosis of COVID-19 exposed persons. Deep artificial neural network algorithms (a subfield of machine learning that are often flagged as AI) has shown striking advances in the ability of machines to interpret large-scale data and make human-level judgments immediately, with minimal cost. These methods have been extensively developed in recent months to diagnose COVID-19 positive patients alongside the RT-PCR testing, using patient self-reported symptoms, clinical data, blood profiles, computerized tomography (CT) scans, and X-ray images. Additionally, AI-based self-diagnosis tools are easily accessible to large population facilitating large-scale pre-screening, and thus help controlling the spread of the virus.

#### AI-assisted self-diagnosis and pre-clinical screening

Because of the unprecedented spread of the pandemic across the individuals in various communities, it is not always feasible to perform RT-PCR-based testing nor the physical screening of all potential patients by the overburdened clinical staff. Accordingly, computational scientists and engineers have developed various nonconventional AI-powered pre-screening tools for COVID-19 infection that can be accessed via smartphone applications. AI/ML-based apps and chatbots utilizes different supervised predictive models that are trained by various symptoms of confirmed cases and designed to identify the COVID-19 infection in patients based on self-reported symptoms. Other apps can pre-screen the patients’ vocal sentiments [51], their cough sounds [52], and breathing patterns [52] using advanced artificial neural network models or monitor vital signs such as oxygen levels, and temperature [53], all recorded via smartphone sensors, via wearable devices or via centralized cameras, microphones, temperature monitors and inertial sensors installed in hospitals and at workplaces.

#### AI-assisted clinical screening

AI models are also used to augment the clinical diagnosis of COVID-19 exposed population. For example, a group of researchers designed unique specific primers using deep learning for ensuring accurate SARS-CoV-2 detection by RT-PCR based platforms [54]. Deep neural networks were frequently applied to assist diagnosis of medical imaging including tomography and X-ray [55, 56] as well as clinical blood sample data [57]. AI-based algorithms discriminate CT scans of COVID-19 associated pneumonia from those with other sources of pneumonias with high specificity in diverse patients (detailed in Additional file 1: Table S2). In multiple studies, AI diagnostic systems achieved equal sensitivity as compared to senior thoracic radiologists for COVID-19 patients. For few instances AI system also corrected the CT scans false negative COVID-19 patients who were then confirmed as positive by RT-PCR showing a clean example of AI-based precise decision making [58]. Investigators selected key clinical and laboratory features to train algorithms to quickly and precisely diagnosis COVID-19 via blood markers such as an elevated alanine aminotransferase, the presence of myalgias, and an elevated hemoglobin [59]. By combining clinical symptoms—e.g., hypertension, low serum albumin, lymphopenia, elevated high-sensitivity C-reactive protein (hsC-RP)—and temporal CT scans, deep learning models were shown to outperform the human-based COVID-19 diagnosis [53]. In another study, neural networks trained on clinical and demographic factors demonstrated 94% accuracy in predicting mortality and long-term hospitalization [60].

## Treatment

Multiple treatment strategies have been used against COVID-19 mainly based on symptomatic therapy and repurposing current antiviral drugs. WHO and NIH recommended interventions such as repurposing broad‐spectrum antivirals (e. g. remdesivir) in combination with anti-inflammatory medicines and interleukin administration (summarized in Additional file 1: Table S1) [16]. Details of current treatment strategies have been detailed in previous reviews and numerous clinical [25] have been designed and registered in the WHO clinical trial platform (https://www.who.int/clinical-trials-registry-platform) to investigate the efficacy of various nominated medicines in different countries [25, 61, 62]. In addition to these strategies, researchers around the world are investigating novel ways for effective and potentially safe medicines to work against the virus or treat the symptoms.

### Neutralizing-based technology

Immunotherapy in both active and passive forms are under clinical trials in many countries to combat against SARS-CoV-2 infection. In active immunotherapy, patient’s immune response is activated or enhanced (e.g., vaccines and direct interferon administration). While in passive immunotherapy, immune molecules are administered to patients who have not produced them on their own (e.g., convalescent plasma and mono or polyclonal antibodies). Different proteins that are involved in the pathogenesis of SARS-CoV-2 such as viral antigens or human immunomodulators, can be targeted for monoclonal antibodies (mAb), mostly full-length IgG-based monoclonal antibody format, and many are under clinical trials [63]. The most common viral targets are epitopes of spike protein that inhibit the attachment of spike proteins to host cell receptors [63–65]. So far, three laboratory-made mAbs (Immunoglobulin G1) against RBD of SARS-CoV-2 spike protein were issued by FDA as EUA. Casirivimab and Imdevimab that should be administered together (by Regeneron Pharmaceutical Inc.) and Bamlanivimb (by Eli Lilly and Company) are recommended for 12 years or older non-hospitalized COVID-19 patients with mild to moderate symptoms, as well as patients aged over 65 or those with certain chronic medical conditions, to prevent the progression to sever stages and hospitalization [24]. Several mAbs were also designed or repurposed targeting human immune responses (e.g., C5A, IL17, IL1β and GM-CSF) and are under clinical trial investigation [63]. Levilimab (Ilsira), (by BIOCAD) and tocilizumab (Actemra) target IL-6 receptor, and itolizumab (by Biocon) targets CD6 [63, 66, 67].

Polyclonal antibodies (pAbs) are of particular interest as they can reduce the risk of possible mutational escape by the virus and decline time, cost, and labor of massive production and clinical investigation of mAbs. Few developed pAbs against COVID-19 are in clinical trial phases including SAB-185 (SAB Biotherapeutics), COVID-HIG and COVID-EIG (Emergent BioSolution) that are from genetically engineered cattle and human plasma-derived immunoglobulins, respectively [63, 68]. Another pAbs from B cells called recombinant anti-coronavirus immunoglobulin (rCIG), GIGA-2050, (GigaGen Inc.) is in the massive production stage and binds to a variety of viral epitopes as claimed by the manufacture [69].

Fusion proteins, nucleic acid-encoding mAbs, nanobodies (single-domain antibodies with small size) and DARPins (designed ankyrin repeat proteins) are other neutralization platforms that are under preclinical or preclinical trails and can target viral proteins, interleukins, or CD molecules [63, 65, 70]. Nanobodies or single heavy-chain antibodies that first discovered in camels and sharks are small proteins with higher solubility and stability and permeability that are widely used and tested for many viral diseases such as HIV, hepatitis B, poliovirus, rabies, etc., [71, 72]. Various nanobodies have been developed and produced against RBD of SARS-CoV-2 spike proteins that are synthetic-based or animal derived-based (llama) [72]. Chi et al. generated five humanized single domain antibodies (sdAbs) from a synthetic library against RBD of SARS-CoV-2 spike protein and neutralization evaluation showed all have inhibitory effect on the SARS-CoV-2 spike pseudotyped particles [73]. In a recent study by Koeing et al. generated multivalent nanobodies targeting spike RBD with more than 100-fold improved neutralizing activity than monovalent nanobodies that is suggested to suppress the emergence of escape mutants [74]. Moreover, a group of researchers reported isolation of alpaca-derived sdAb, named Ty1, that targets RBD of spike protein in SARS-COV-2 with high affinity and preventing its attachment to ACE2 [75]. Gai et al. immunized four camels with RBD of SARS-CoV-2 spike protein and identified 381 nanobodies in the plasma that can recognize it. Seven nanobodies were able to block the RBD-ACE2 interaction of which NB11-59 had highest activity against the virus [76]. They suggested a large-scale production and an inhaled delivery of NB11-59 [76].

DARPins, designed ankyrin repeat proteins, are small size protein scaffolds with simple architecture of one protein with one domain. DARPine molecules can be designed as mono or multi-specific single chain molecules linked by peptide linker and, therefore, these multi-DARPines can attach to more than one epitope at once. Walser et al*.* have developed mono and multi-DARPins against spike protein of SARS-CoV-2 and in vivo studies on hamster showed significant reduction of virus pathogenesis [77]. Multi-DARPin molecules including MP0420 and MP0423, combining of three independent DARPin domains, binding specifically to SARS-CoV-2 RBD or other part of spike protein respectively, are designed of which the efficacy of ensovibep (MP0420) is under investigation in a clinical trial [63, 70, 78]. In vitro studies showed that these two multi-DARPins are highly potent against the new circulating SARS-CoV-2 variants including UK variant (B.1.1.7) and South African variant (B.1.351) [78].

### CRISPR-based technology

Beyond a diagnostic technique, CRISPR-cas gene editing technology is also suggested as a therapeutic approach against COVID-19. Recently, Abbot et al. [79] were investigated the genome cleavage and degradation of SARS-CoV-2 and other coronaviruses using RNA-guided endonucleases (Cas13d) and crRNA. They developed a PAC-MAN system (Prophylactic Antiviral CRISPR in Human cells) and designed multiple crRNAs targeting SARS-CoV-2. They reported 85% and 70% repression of signal reporters fused to RdRP protein and N protein respectively in human lung epithelial cell line. Lipofection, electroporation, nucleofection, microinjection, and viral vectors are some approaches for transferring CRISPR-system into the targeted cells [80]. For instance, antibody and CAS (ABCAS) fusion approach was used to deliver the complex of cas13-antibody that is specific to the S protein of SARS-CoV-2 and selectively deliver it only to the infected host cell [81].

### Nano-based technology

Nano-based technologies for drug encapsulation and drug delivery to the infected cells have been investigated for the treatment of viral infections such as influenza, HIV, HBV (Hepatitis B virus) or HCV (Hepatitis C virus) to increase the specificity and efficacy of the antivirals and various nanomedicines [82, 83]. Dexamethasone nanomedicine that is used previously for treatment of rheumatoid arthritis, inflammatory bowel disease and multiple sclerosis was proposed to administer for COVID-19 patients [84]. Lipid nanoparticles as delivering particles (NLPs) were used by Imperial College London and Arcturus Therapeutics to design and develop encapsulated self-replicating mRNA vaccines [85]. Zhang et al. [86] developed cellular nanosponges using plasma membrane of pulmonary type II epithelia cells or human macrophages. The nanosponges attract and neutralize SARS-CoV-2 by presenting ACE2 on their surfaces that binds to spike protein to prevent or reduce the infection of other cells. The implementation of theranostic nanoparticles that can combine and carry therapeutics to the targeted cells via intranasal delivery was also proposed [87].

### AI-assisted and network-based drug repurposing

In addition to helping in early diagnosis and identifying patients at risk of clinical deterioration and poor outcomes, artificial intelligence and machine learning have been applied extensively to accelerate the development of new treatments effective against SARS-CoV-2. Deep artificial neural networks (i.e., deep learning) allow to predict the molecular structure of proteins crucial for viral entry/replication and can also come in handy to virtually visualize the interactions of the target with ligands and therapeutic compounds, the information essential for de novo drug discovery [88–90].

Advanced ML/AI algorithms and bioinformatics pipelines implemented on high-performance computing platforms have generated the capacity to simultaneously read complex viral and host genomic and protein sequences, identify similarities of SARS-CoV-2 pathogenic factors to known viruses, analyze preclinical/clinical research data, and integrate all this information to learn potential causal interactions and extract key features to screen drugs/compounds from the available drug repertoire paving the way for drug development in a concise time [91].

Multiple researchers used machine learning methods to accelerate the drug discovery program against COVID-19 [92–94]. Deep learning-based algorithms have helped designing new molecules that could terminate SARS-CoV-2 replication [95] and identified 10 potential compounds from a list of 4895 drugs [96]. A deep residual networks is employed by Google Deep for predicting protein structures of SARS-COV-2 membrane proteins [97]. DeepTracer is also a program based on customized deep convolutional neural network which developed protein structure of SARS-CoV-2 from high-resolution cryoelectron microscopy density maps and amino acid sequences [98]. A recent study has reviewed the role of AI for accelerating COVID-19 drug repurposing and justified that incorporation of AI approaches into drug discovery pipeline is not just formidable but necessary [99].

Besides innovative AI algorithms, the emerging field of network medicine quantifies the relationship between the virus–host interactome and drug-host and viral-drug network, to estimate drug-disease proximity and reveal targets for repositioning existing drugs against COVID-19 [100–102]. Accordingly, multiple drug repurposing tools such as CoV-KGE [103], COVID-CDR [104], and COVEX [105] have been developed to investigate potential individual and combination repurposed compounds of potential efficacy against COVID 19. Network-based in silico analysis, for instance, has suggested the potential role of toremifene in blocking the interaction between ACE2 and the spike protein of SARS-CoV-2 and inhibiting non-structural protein 14 [106]. As another example, Zhou et al*.* used network medicine methodologies combined with large-scale patient clinical and multi-omics data to understand SARS-CoV-2 pathogenesis and potential therapies [107]. They identified that melatonin intake is significantly associated with a reduced likelihood of a positive laboratory test result for SARS-CoV-2 confirmed by RT-PCR assay [107]. Beyond these few examples, a recent review has discussed multitude of network-based drug repositioning approaches applicable to COVID-19 [108].

Beyond in silico approaches, large-scale in vitro screening of approved chemical compounds and natural products can guide drug repositioning strategies and speed up prioritization of repositioning candidates for clinical investigation. For instance, Riva et al*.* [109] have profiled over 12,000 clinical-stage or FDA-approved small molecules to identify existing drugs that harbor antiviral activity against SARS-CoV-2 in a cell-based assay. They found 30 known drugs that inhibit viral replication of which six can be used with effective therapeutic dose. In another study, Ellinger et al*.* [110] screened 5632 compounds using human epithelial colorectal adenocarcinoma cell line and identified 64 compounds with inhibition effect on viral-induced cytotoxicity. More recently, Chen et al*.* [111] carried out a high-throughput screening with compound collection of 8810 approved and investigational drugs, mechanism-based bioactive compounds, and natural products using a SARS-CoV-2 cytopathic assay. They identified 319 compounds with anti-SARS-CoV-2 activities including 91 approved and 49 investigational drugs.

### Complementary medicines

Complementary medicines including herbal medicines have been widely applied all around the world since the COVID-19 outbreak. In China, the official Diagnosis and Treatment Protocol for COVID-19 recommends Traditional Chinese Medicine (TCM) as a complementary intervention against COVID-19 according to the results observed during SARS pandemic in 2002 [112, 113].

Curcumin, as an active constituent of *Curcuma longa* (turmeric), has been suggested to modulate the increased rate of inflammatory cytokines and its anti-inflammatory effects can be due to its effect on eicosanoid biosynthesis [114]. Curcumin is suggested to alleviate the inflammatory cytokines in COVID-19, which may cause an improvement in clinical manifestation and overall recovery [115]. In a double-blind clinical trial, nano particles of curcumin have been evaluated on inflammatory biomarkers in COVID-19 patients. The results showed that nano-curcumin can reduce IL-6 and IL-1β gene expression and secretion in serum. However, it did not make significant changes in IL-18 mRNA expression and TNF-α concentration.

Thyme (*Thymus vulgaris*) has been recommended by Traditional Persian Medicine for the treatment of respiratory diseases due to its antioxidant and antiviral properties. In a randomized controlled clinical trial, thyme essential oil was evaluated for its effects on the symptoms. The results revealed that after 1 week of thyme administration, a number of symptoms such as muscular pain, headache, dizziness, cough, chest wall pain and lethargy were significantly reduced in the treatment group compared to the control group [116].

Several complex herbal medicines have been used in clinical research to treat COVID-19 symptoms. *Xuanfei Baidu* decoction, *Qingfei Touxie Fuzhen*, *Toujie Quwen* granules, *Reyanning mixture*, *Shufeng Jiedu* and *Lianhua Qingwen* capsules are among Chinese complex herbal medicine which are explored in clinical trials. Recently, a systematic review and meta-analysis, evaluated 18 clinical trials on Chinese herbal medicines (CHM) for COVID-19 involving 2275 patients. The most prevalent herbs used in clinical trials were Liquorice root (*Glycyrrhizae glabra*), Baical Skullcap root (*Scutellariae baicalensis*), Pinellia rhizome (*Pinelliae Tematae*), Bitter Apricot seed (*Prunus armeniaca*) and Forsythia fruit (*Forsythiae Suspensae*). The results of meta-analysis revealed that CHM-treated group compared to conventional western medicine-treated group has shown the improvement of 13 standard clinical parameters in CHM-treated patients such as clinical cure rate, lung CT, length of hospital stay, fever level and reduction rate, and inflammatory biomarkers (C-reactive protein) among others. Moreover, no severe adverse effects were identified by CHM [117]. Notably, Lianhua Qingwen (LHQW) capsule which contains 11 herbs was approved by China National Medical Products Administration as an alternative treatment improving the symptoms of the COVID-19 disease through regulating inflammatory responses and improving the function of immune system [113]. Quercetin, luteolin, kaempferol, baicalein, isorhamnetin, wogonin, and naringenin are among the main active ingredients of Chinese medicines for alleviating the symptoms of COVID-19 by targeting inflammatory mediators, eliminating free radicals, and regulating immune system [118]. Additionally, natural products such as dihydrotanshinone, gallic acid, emodin and quercetin are potentially effective for the prevention of COVID-19. Research on these molecules is still in the preclinical stage [119].

Acupuncture, an integral part of TCM, may play a role in the treatment of breathlessness in COVID-19 [120]. Acupuncture has been widely assessed by various randomized controlled trials (RTCs) for the treatment of respiratory disorders and now its role in COVID-19 treatment is under investigation in clinical trials [121, 122]. The results of a recent systematic review and meta-analysis, including 12 studies with 597 patients, revealed that acupuncture can relieve breathlessness in patients with advanced non-malignant diseases [123].

### Prevention

Vaccination is the most effective preventative measure against infectious diseases. During 2020, substantive collaborations have been shaped among multinational pharmaceutical industries, national governments, biotechnology companies, and research institutes focusing on a COVID-19 vaccine development [124]. Various development and manufacturing platforms (Table2)  have been used for vaccine production. However, major current vaccines that are in the clinical trial phases are either derived from inactivated virus platforms or, in most cases, are based on new recombinant technologies such as non-replicating viral vectors, RNA-based vaccines, recombinant protein subunit and virus like particles. According to the latest WHO draft landscape of COVID-19 candidate vaccines (June 22nd 2021), 103 vaccines are in clinical phase, of which 32% use protein subunit platform. Viral vector (non-replicating), RNA and inactivated platforms each with 16% are in the second place. Five and 29 vaccines are in the 4th and 3rd phase of clinical trial respectively, of which 16 are approved or given approval for emergency immunization in various countries. We developed an online dashboard (http://vafaeelab.com/COVID19_vaccine.html) to provide up to date information on clinically approved vaccines or those in the latest phase of clinical trials.

**Table 2:** Summary of platforms used for developing and manufacturing vaccines for COVID-19

Vaccine platform	Technology	Advantages	Disadvantages	References
Inactivated virus	Heat/radiation/chemical inactivation of replicated viruses in cell lines	Well established technology, contains whole genome and proteins of virus for immunogenicity, no chance of infection	Slow production in cell lines, adverse effects due to adjuvant or virus components, BCL3 facilities, high quality control, different cell lines and related issues, several boosting injections for longer immunity	[138, 139]
Live attenuated	Weakened modified replicated viruses in cell lines	Well established technology, strong long-term immunity, contains whole genome and proteins of virus for immunogenicity	Slow production in cell lines, BCL3 facilities, Risk of infection in individuals, possible pathogen polymorphism inside host, safety concerns for risk groups	[138, 139]
Protein subunits	Components of purified viral antigens produced by recombinant technology	Safe with less adverse effects, no BCL3 facilities for virus replication, non-infection, strong humoral response	Limited selection of Ag and partial protection, Ag adaptation in the pathogen for better fitness in host, need adjuvant for better immunisation, poor induction of cellular responses, no cellular immune response	[138, 139]
Virus like particles (VLP)	Complex of several viral proteins that have ability to self-assemble when recombinantly expressed in various bacterial or yeast platforms without having the viral genome produced	Non-infection, strong humoral responses, do not need adjuvant, optimal size to be absorbed by antigen presenting cells (APC)	Complex manufacturing process, stability issues, impurities during production, side effects of expression systems	[140, 141]
Viral vector (replicating or non-replicating)	Integration of target genes into another harmless viral genomes (mostly adenoviruses) as a carrier and then the target gene is expressed by the host cells	Non-infection induction of T and B cell immune response, long term gene expression	Potential risk of vector and related adverse effects, reduced efficacy in case of pre-existing vector immunity, induction of vector immunity rather than target virus, time and cost due to cell line-based production	[142, 143]
DNA-based	Integration of target gene into plasmid as a carrier and then the target gene is expressed by the host cells	Induction of T and B cell immune response, non-infectious, no BCL3 facilities for virus replication rapid, egg and cell line free, cost and time effective, stable vaccine for transportation	Potential integration into human genome, poor immunogenicity in human, not enough data for safety and efficacy	[138, 139]
Conventional mRNA (non-replicating mRNA, NRM)	Synthetic mRNA (flanked by 5′ and 3′ untranslated regions (UTRs), a 5′-cap structure and a 3′-poly-(A) tail) of target gene encapsulated in synthetic lipid or polymer carrier as a carrier and then the target gene is expressed by the host cells	Rapid scale production, cell line free, no BCL3 facilities, egg and cell line free, strong T cell response, non-infection	Not enough data for safety and efficacy, higher dose of RNA compared to SAM, possible degradation of mRNA in the host cells leading decline in vaccine potency	[127, 144]
Replicon (self-replicating mRNA, SAM)	Auto-replicative activity by adding a large open reading frame for four non-structural proteins and sub-genomic promoter at the 5′ end and encapsulated in the lipid as a carrier and then the target gene can be amplified by itself and then expressed by the host cells	Rapid scale production, cell line free, no need for BCL3 facilities, egg and cell line free, lower dose of RNA compares to RNM due to self-replicative properties, induction of T and B cell immune response	Not enough data for safety and efficacy, larger sequence size and more complicated design compared to SAM since it needs replicons for self-amplifying activity, possible degradation of mRNA in the host cells leading decline in vaccine potency	[127, 144]
Plant-based (edible vaccines)	Integration of antigen gene in the genome of	Large scale production, no adverse effects due to injection, time and cost effective for large production, no adjuvants or harmful components, no need for cold chain transportation and delivery, not need high end and BCL3 facilities for production	Consistency of dosage in plants and individuals, vaccine dosage might be variable due to the size of plants, instability during food preparation, not convenient for infants	[132, 133]

CoronaVac (Sinovac Research and Development Co) and BBIBP-CorV (Sinopharm Co) are two Chinese inactivated vaccines that used largely in different countries during the clinical trials and approved by some countries such as China, UAE and Indonesia.

Nano-based vaccine are also of interest and recently, nucleic acid-based platforms have been developed where synthetic sequences are used to express proteins including microbial Ag in the cell and induce humoral and cell-mediated immunity. These includes DNA and RNA based vaccines delivered to the body by several platforms. Synthetic mRNAs, including conventional (non-amplifying) mRNA and self-amplifying mRNA (saRNA or replicons with an ORF for four non-structural proteins at the 5′ end), have been investigated in recent years in clinical trials against infectious diseases [125]. While antigen expression depends on the number of successfully delivered conventional RNAs to the host cells, the replicative activity of the saRNA vaccines increases their efficacy due to the lower dose for vaccination [126, 127]. So far, two nano-conventional mRNA vaccines known as BNT16b1, (BioNTech, Fosun Pharma, and Pfizer) and mRNA1273 (Moderna Co.) consisting of a modified mRNA of SARS-CoV-2 spike protein have been received primary approval and emergency usage approval in some countries [128, 129].

Adenoviruses can be effectively used as a vector to introduce foreign DNA into the target cells and induce host immune responses and therefore used in new developed vaccines against human and animal pathogens such as HIV, influenza, rabies and ebola [130]. Sputnic V (Gamaleya Research institute), AZD1222 (Oxford University and AstraZeneca), and Ad26.COV2.S (Janssen pharmaceuticals) as three vaccines based on Adenovirus non-replicating viral vectors have also been approved in Russia and UK and Bahrain respectively [128].

Apart from current technologies and platforms to develop and manufacture vaccines, there are some other approaches that can be used or are under investigation for vaccine development. For instance, edible or plant-based vaccine as a platform for bulk production of vaccines is of interest. In this approach the desire gene of proteins or antigens are incorporated into the plants (such as tobacco, turnip, and potato) without losing its immunogenicity [131]. Plants then can be commercially cultivated on a large scale as cost and time saving platform in some high demand situations like what we encounter for COVID-19 now. This approach has been developed to produce biological products such as vaccines, antibodies, immunomodulatory proteins, drugs and biologicals in a large scale and the first approved edible vaccine is manufactured for Newcastle disease in chickens in 2006 [132] and several clinical trials are investigating the efficacy and safety of medicines for treatment of chronic and infectious diseases [133]. Zheng et al., investigated immunogenicity of tobacco-expressed SARS-CoV nucleocapsid protein in mice [134]. Currently several companies such as Kentucky BioProcessing company, British and American Tobacco Company, Medicago Inc. and iBio are investigating the development of tobacco-based SARS-Cov-2 protein subunit or virus like particle (VLP) vaccines [133]. *Saccharomyces cerevisiae* has been also used for the development of edible vaccine that exploit spike, envelope and membrane proteins [135].

## Conclusion

Since 2019, the COVID-19 pandemic has raised several public health and economic concerns across the globe. It has challenged policymakers, and health experts, as well as communities and populations to overcome the situation and combat the disease using numerous applicable approaches. It also opened a new window for scientists and researchers in various fields to develop new tools using interdisciplinary approaches for detection, treatment, and prevention of Covid-19 of which some have been commercially used and helped frontline health workers to conquer the disease. In this review, we discussed some approaches that are developed for faster and more reliable detection of COVID-19 such as CRISPR-based, Nano-based, mass spectrometry, and AI-based models. Furthermore, we reviewed selective drug repurposing strategies based on deep leaning and network pharmacology approaches as well as therapeutic approaches based on CRISPR-cas and Nano technologies, neutralizing factors such as mono/polyclonal antibodies and DARPins. Beyond these emerging therapeutic approaches, we reviewed some clinically-evaluated complementary medicines that help to relief the symptoms. Moreover, current vaccine platforms and some approved vaccines against the disease were discussed and a dashboard were designed to update current vaccines used in different countries.

Although the spread of SARS-CoV-2 and resulted infection, COVID-19 has huge global social, financial and health impacts, it provided an opportunity for scientist and researchers in different fields for innovative approaches and showed they can develop potential valid tools that playmakers can rely on to manage the pandemic. This can be a global model for unpredictable situations that may happen in the future.

## Funding and acknowledgments

This study received no external funding.

## Supplementary Information

**Additional file 1: Table S1.** Current treatment strategies for COVID-19 patients recommended by the USA National institute of Health (NIH). **Table S2.** Selected studies describing the use of AI and ML in COVID-19 diagnosis and disease predictions.

## Data Availability

Not applicable.
