# Supplementary material for: An update on novel approaches for diagnosis and treatment of SARS-CoV-2 infection
Source: Cell Biosci. 2021 Aug 22;11:164. doi: 10.1186/s13578-021-00674-6 (PMC8380468; doi:10.1186/s13578-021-00674-6)
Supplement: Supplementary file 1 — Additional file 1: Table S1. Current treatment strategies for COVID-19 patients recommended by the USA National institute of Health (NIH). Table S2. Selected studies describing the use of AI and ML in COVID-19 diagnosis and disease predictions. [file 13578_2021_674_MOESM1_ESM.docx]

**Additional file 1**

**Table S1:** Current treatment strategies for COVID-19 patients recommended by the USA National institute of Health (NIH). Information is compiled from NIH COVID-19 Treatment Guidelines (<https://www.covid19treatmentguidelines.nih.gov/>), June 27, 2021 update.

| **Treatment** | **Approach** | **Examples** | **Mechanism of action** | **Recommended for** |
| --- | --- | --- | --- | --- |
| Respiratory support | Oxygen therapy | Nasal cannula/simple face mask | Increase Oxygen level | Regular hospital/ home care |
|  |  | High flow/reservoir mask | Increase Oxygen level | Hospital mild to severe care |
|  |  | Continuous Positive Airway Pressure (CPAP) machine | Increase Oxygen level | Hospital use for patients with apnoea or to open airways |
|  |  | Ventilators | Increase Oxygen level | In acute respiratory failure / ICU situation |
| Antivirals | Antiviral activity | Remdesivir | Interact with RNA dependant RNA polymerase (RdRp) to inhibit viral replication, previously used for Ebola | Hospitalized patients with mild cases of COVID-19 |
| Viral Neutralizer | SARS-CoV-2 Mono/poly clonal antibody approach | COVID-19 Convalescent Plasma | Polyclonal antibodies from Covid-19 positive patients to neutralise virus in early stage of infection | Hospitalizes >18 patients in early stage of the infection |
|  |  | REGN-Cov2 (Regeneron)  (a combination of casirivimab and imdevimab) | Inhibits entrance of virus to host cell by attachment to RBD of spike protein | High-risk outpatients with mild to moderate COVID-19 |
|  |  | Bamlanivimab (LY-CoV555) + Etesvimab | Inhibits entrance of virus to host cell by attachment to RBD of spike protein | High-risk outpatients with mild to moderate COVID-19 |
| Immuno-modulators | Anti-inflammatory | Baricitinib in combination with remdesivir | Prevent the dysregulated production of proinflammatory cytokines typically observed in people with Covid‐19 | Hospitalized patients on high-flow oxygen who have evidence of clinical progression or increased markers of inflammation. |
|  |  | Dexamethasone/prednisolone/hydrocortisone | Corticosteroids | Hospitalised patients who require supplemental oxygen therapy |
|  |  | Tocilizumab with dexamethasone | Inflammatory signal Blockers (mAb as IL6 inhibitor) | For most hospitalized patients with hypoxemia who require conventional oxygen therapy |
|  |  | Kevzara (Sarilumb) | mAb (IL6 receptors) |  |
|  |  | Colchicine | Interfering with cells involved in inflammation | Non-hospitalized patients in early stage of infection |
|  | Interferons | IFN- alfa-2a | Viral RNA degradation, RNA transcription alteration, protein syn- thesis inhibition and apoptosis | High-risk outpatients with mild to moderate COVID-19 |
|  |  | IFN- alfa-2b | Viral RNA degradation, RNA transcription alteration, protein syn- thesis inhibition and apoptosis | High-risk outpatients with mild to moderate COVID-19 |
|  |  | INF-βeta | Viral RNA degradation, RNA transcription alteration, protein syn- thesis inhibition and apoptosis | High-risk outpatients with mild to moderate COVID-19 |
|  | Interleukin-1 inhibitor | Anakinra | Blocks the activity of both IL-1α and IL‑1β | There are insufficient data to recommend for or against the use of Anakinra in COVID-19 patients, however it can be used for patients in ICU to lower the risk of need for mechanical ventilation |
|  | Interleukin-6 inhibitor | Kevzara (Sarilumab) | Inflammatory signal Blockers (as IL6 inhibitor) | For patients who do not require ICU care or are admitted to the ICU for >24 hours |
|  |  | Tocilizumb | Inflammatory signal Blockers (as IL6 inhibitor) | In combination with dexamethasone in certain hospitalized patients who are exhibiting rapid respiratory decompensation due to COVID-19. |

**Table S2**: Selected studies describing the use of AI and ML in COVID-19 diagnosis and disease predictions.

|  | **Publication** | **ML/AI method** | **Types of data** | **No of patients** | **Validation method** | **Accuracy** |
| --- | --- | --- | --- | --- | --- | --- |
| Image-based Diagnosis (Deep Learning) | Jin, Cheng et al.*,* [1] | Slice diagnosis module- a 2D deep convolutional neural network based on ResNet-101 | Chest CT scans | 11,356 CT images of 9025 subjects consisting of COVID-19, non-viral community-acquired pneumonia, influenza, and non-pneumonia Patients | Cross Validation | Sensitivity and specificity for COVID-19 were 87.03 and 96.60, and the multi-way Accuracy: 97.81 % |
|  | Han, Z. *et al.,* [2] | Attention-Based Deep 3D Multiple Instance Learning  convolution neural networks | Chest CT scans | 79,100,130; 79 patients with COVID-19, 100 CT examples from 100 patients with common pneumonia, and 130 CT examples from 130 people without pneumonia | Cross Validation | Overall accuracy of 97.9%, AUC of 99.0%, and Cohen kappa score of 95.7% |
|  | Zhang, K. *et al.* [3] | Lesion segmentation, diagnosis prediction model | Chest CT scans | 4154; A total of 617,775 CT slices of 6752 CT scans from 4154 patients | Cross validation | AUC of 97.97% |
|  | Ardakani, A. A *et al.,*[4] | Deep Convolutional Neural Network ResNet-101 | Clinical, Mammographic | 1020 CT images of 108 volume of patients with laboratory confirmed Covid-19, 86 CT images of viral and atypical pneumonia patients, | Holdout | Accuracy: 99.51% Specificity: 99.02% |
|  | Li *et al.,* [5] | Deep learning model | Clinical, Mammographic | 4352 chest CT scans from 3322 patients | Cross-validation | Accuracy: 96% Specificity: 98.00% |
|  | Ouyang *et al.,* [6] | 3D convolutional network (CNN) | Chest CT scan | 1588, we collect 2186 CT scans from 1588 patients for a 5-fold cross-validation | Cross validation | AUC value: 94.4, Accuracy: 87.5%, Sensitivity of 86.9%, Specificity of 90.1%, and F1-score of 82.0%. |
|  | Ozturk, T. *et al.,* [7] | Convolutional Neural Network Dark Covid Net Architecture | Clinical, Mammographic | 127, 43 f, 82 m 500, 500; 127 X-ray images with 43 female and 82 male positive cases 500 no-findings and pneumonia cases of 500 | Cross-validation | Accuracy: 98.08% on Binary classes  Accuracy: 87.02% on Multi-classes |
|  | Sun, L *et al.,* [8] | Support Vector Machine | Clinical, laboratory features, Demographics | 336, 220; 336 infected patients with PCR kit, 26 severe/critical cases and 310 non-serious cases and with another related disease79 hypertension, 29 diabetes, 17 coronary disease and 7 having history of tuberculosis | Holdout | Totally, 36 clinical indicators significantly associated with severe/critical symptom were identified. Accuracy: 77.5% Specificity: 78.4% AUROC reaches 0.99 training and 0.98 testing dataset |
| Non-image Diagnosis (Traditional Machine Learning) | Wu, J. *et al.,* [9] | Random forest Algorithm | Clinical, Demographics | Total of 253 samples from 169 patients suspected with Covid-19 collected from multiple sources. Clinical blood test of 49 patients derived from commercial clinic center. 24 samples infected patient with Covid-19 | Cross-validation | Accuracy: 95.95% Specificity: 96.95%  11 key blood indices were extracted |
|  | Ribeiro, M. H. D. M., *et al.,* [10] | Support Vector Regression and stacking-ensemble | Clinical | 40.581 | Holdout | Accuracy: Error in range of 0.87%-3.51% one, 1 .02%–5.63% three and 0.95% -6.90% six day ahead |
|  | Yan, L. *et al.*, [11] | XGBoost classifier | Clinical, Blood samples of 75 features | 485 | Cross-validation | Accuracy: 90% |
|  | Chimmula, V.K.R., *et al.,* [12, 13] | Deep Learning using LSTM network | Demographic | John Hopkins University & Canadian Health authority, data containing infected cases up to March 31, 2020 | Cross-validation | Ending point of the pandemic outbreak in Canada was predicted on June 2020 |
|  | Chakraborty, T. and Ghosh, I. [[40]](https://www-sciencedirect-com.wwwproxy1.library.unsw.edu.au/science/article/pii/S0960077920304562#bib0040) | Hybrid Wavelet- autoregressive integrated moving average model and regression tree | Demographic | India: 64 UK: 65 Canada:70 France: 71 South Korea: 76 | Cross-validation | Real-time forecast and 10 days ahead, observed seven key features associated with dead rate. |
|  | Chansik, An *et al.,* [14] | Cox proportional hazards regression analysis, the least absolute shrinkage and selection operator (LASSO), linear support vector machine (SVM), SVM with radial basis function kernel, random forest (RF), and k-nearest neighbors were tested | sociodemographic and medical information | South Korea 10,237 COVID-19 patients | Cross-validation | most significant predictors for LASSO included old age and pre-existing DM or cancer; for RF they were old age, infection route (cluster infection or infection from personal contact), and underlying hypertension |

**References**

1. Jin, C., *et al.,* Development *and evaluation of an artificial intelligence system for COVID-19 diagnosis.* Nat Commun, 2020. 11(1): p. 5088.

2. Han, Z., *et al., Accurate Screening of COVID-19 Using Attention-Based Deep 3D Multiple Instance Learning.* IEEE Trans Med Imaging, 2020. 39(8): p. 2584-2594.

3. Zhang, K., *et al., Clinically Applicable AI System for Accurate Diagnosis, Quantitative Measurements, and Prognosis of COVID-19 Pneumonia Using Computed Tomography.* Cell, 2020. 182(5): p. 1360.

4. Ardakani, A.A., *et al.* *Application of deep learning technique to manage COVID-19 in routine clinical practice using CT images: Results of 10 convolutional neural networks.* Comput Biol Med, 2020. 121: p. 103795.

5. Li, L., *et al.,* *Using Artificial Intelligence to Detect COVID-19 and Community-acquired Pneumonia Based on Pulmonary CT: Evaluation of the Diagnostic Accuracy.* Radiology, 2020. 296(2): p. E65-E71.

6. Ouyang, X., *et al., Dual-Sampling Attention Network for Diagnosis of COVID-19 From Community Acquired Pneumonia.* IEEE Trans Med Imaging, 2020. 39(8): p. 2595-2605.

7. Ozturk, T., *et al., Automated detection of COVID-19 cases using deep neural networks with X-ray images.* Comput Biol Med, 2020. 121: p. 103792.

8. Sun, L., *et al., Combination of four clinical indicators predicts the severe/critical symptom of patients infected COVID-19.* J Clin Virol, 2020. 128: p. 104431.

9. Wu, J., Zhang, P., Zhang, L., Meng, W., Li, J., Tong, C., Li, Y., Cai, J., Yang, Z., JZhu, J., Zhao, M., Huang, H., Xie, X. and Li, S., *Rapid and accurate identification of COVID-19 infection through machine learning based on clinical available blood test results.* medRxiv, 2020.

10. BBC. *Coronavirus: Israel enables emergency spy powers*. 2020 30 Dec 2020]; Available from: <https://www.bbc.com/news/technology-51930681> (

11. Ribeiro, M., *et al., Short-term forecasting COVID-19 cumulative confirmed cases: Perspectives for Brazil.* Chaos Solitons Fractals, 2020. 135: p. 109853.

12. Torres-Macho, J., *et al., The PANDEMYC Score. An Easily Applicable and Interpretable Model for Predicting Mortality Associated With COVID-19.* J Clin Med, 2020. 9(10).

13. Pan, P., *et al., Prognostic Assessment of COVID-19 in the Intensive Care Unit by Machine Learning Methods: Model Development and Validation.* J Med Internet Res, 2020. 22(11): p. e23128.

14. An, C., *et al., Machine learning prediction for mortality of patients diagnosed with COVID-19: a nationwide Korean cohort study.* Sci Rep, 2020. 10(1): p. 18716.
